# Correction: The role of perceived autonomy support and fear of failure: A weekly diary study on work-related rumination

**DOI:** 10.1371/journal.pone.0298248

**Published:** 2024-01-31

**Authors:** Elif Manuoglu

## Notice of Republication

An incorrect version of S1 File and S2 File was published in error. This article was republished on January 18, 2024, to correct for this error. Please download this article again to view the correct version.
